# Advanced abdominal imaging with dual energy CT is feasible without increasing radiation dose

**DOI:** 10.1186/s40644-016-0073-5

**Published:** 2016-06-21

**Authors:** Monika Uhrig, David Simons, Marc Kachelrieß, Francesco Pisana, Stefan Kuchenbecker, Heinz-Peter Schlemmer

**Affiliations:** Department of Radiology, German Cancer Research Center (DKFZ) Heidelberg, Im Neuenheimer Feld 280, D-69120 Heidelberg, Germany; Department of Medical Physics in Oncology, Division of X-Ray Imaging and Computed Tomography, German Cancer Research Center (DKFZ) Heidelberg, Im Neuenheimer Feld 280, D-69120 Heidelberg, Germany

**Keywords:** Dual energy CT, Radiation dose, Abdominal imaging, Oncological imaging

## Abstract

**Background:**

Dual energy CT (DECT) has proven its potential in oncological imaging. Considering the repeated follow-up examinations, radiation dose should not exceed conventional single energy CT (SECT). Comparison studies on the same scanner with a large number of patients, considering patient geometries and image quality, and exploiting full potential of SECT dose reduction are rare. Purpose of this retrospective study was to compare dose of dual source DECT versus dose-optimized SECT abdominal imaging in clinical routine.

**Methods:**

One hundred patients (62y (±14)) had either contrast-enhanced SECT including automatic voltage control (44) or DECT (56). CT dose index (CTDIvol), size-specific dose-estimate (SSDE) and dose-length product (DLP) were reported. Image noise (SD) was recorded as mean of three ROIs placed in subcutaneous fat and normalized to dose by $SDn=SD\times \sqrt{CDTIvol}$*.* For dose-normalized contrast-to-noise ratio (CNRD), mean attenuation of psoas muscle (CT_muscle_) and subcutaneous fat (CT_fat_) were compared by *CNRD* = (*CTmuscle* − *CTfat*)/*SDn*. Statistical significance was tested with two-sided *t*-test (α = 0.05).

**Results:**

There was no significant difference (*p* < 0.05) between DECT and SECT: Mean CTDIvol was 14.2 mGy (±3.9) (DECT) and 14.3 mGy (±4.5) (SECT). Mean DLP was 680 mGy*cm (±220) (DECT) and 665 mGy*cm (±231) (SECT). Mean SSDE was 15.7 mGy (±1.9) (DECT) and 16.1 mGy (±2.5) (SECT). Mean SDn was 42.2 (±13.9) HU $*\sqrt{\mathrm{mGy}}$ (DECT) and 47.8 (±14.9) HU $*\sqrt{\mathrm{mGy}}$ (SECT). Mean CNRD was 3.9 (±1.3) ${\mathrm{mGy}}^{-\frac{1}{2}}$. (DECT) and 4.0 (±1.3) ${\mathrm{mGy}}^{-\frac{1}{2}}$ (SECT).

**Conclusion:**

Abdominal DECT is feasible without increasing radiation dose or deteriorating image quality, even compared to dose-optimized SECT including automatic voltage control. Thus DECT can contribute to sophisticated oncological imaging without dose penalty.

## Background

Dual energy computed tomography (DECT) is an exciting development in CT technology and has multiple clinical benefits [1–4]. Applications include characterization of renal stones [5–7], visualization of lung perfused blood volume and ventilation [8–11] as well as assessment of myocardial perfused blood volume [12–14]. In oncological imaging, DECT has proven potential for detection and characterization of liver and kidney masses [15, 16], characterization of pulmonary nodules [17] and therapy monitoring [18–20].

Considering that oncological patients have repeated follow-up examinations, dose issues should not be neglected. It is suggested that about 2 % of all cancers in the US are caused by radiation exposure from CT [21]. Some authors make attempts to predict the effect of radiation dose, e.g., that 29 000 patients will have cancer due to the CT examinations performed 2007 in US [22]. Another study estimates, that 1 of 270 women getting a CT of the coronary arteries will suffer from cancer caused by this examination [23]. In this context, convincing evidence is needed proving that advanced DECT applications do not require increased radiation exposure.

There are previous studies and reviews comparing dose of DECT and conventional single energy CT (SECT) [24–30] for different examination types. However, studies in clinical routine with a large number of patients, taking into account patient geometries and image quality, on the same scanner and exploiting the full potential of dose reduction of SECT including automatic voltage control are rare. Results of previous investigations are still controversial. Current studies indicate a potential compromise of image quality for DECT, in contrast to many previous investigations suggesting that DECT offers comparable signal to noise ratios when compared to SECT [31]. This demonstrates the need of further studies evaluating DECT dose efficiency, especially in clinical routine.

The purpose of this study was to compare radiation dose of abdominal DECT versus dose-optimized SECT including tube current and voltage modulation on the identical dual source CT in clinical routine, considering patient geometries and image quality.

## Methods

### Patient population

This retrospective study was approved by local ethics committee (Ethics committee of Medical Faculty of Heidelberg), and informed consent was obtained from all patients.

Over a period of 6 weeks (March 2015–April 2015) 100 CT scans (44 SECT, 56 DECT) of 100 oncological patients were evaluated (mean age, weight, height and body mass index are listed in Table 1).

**Table 1:** Patient population

	Number of patients	Age (y)	Weight (kg)	Height (cm)	BMI^a^ (kg/m^2^)	Lateral diameter (cm)
All	100	61.6 (±13.6)	80.7 (±17.1)	173.2 (±10.4)	26.8 (±4.9)	36.9 (±5.3)
DECT^b^	56	60.3 (±12.8)	83.9 (±18.0)	175.9 (±10.3)	27.0 (±4.8)	37.2 (±5.5)
SECT^c^	44	63.2 (±14.6)	76.6 (±15.2)	169.9 (±9.5)	26.6 (±5.1)	36.6 (±5.1)

Values are given as means (± standard deviation)

^a^body mass index; ^b^dual energy CT; ^c^single energy CT

Inclusion criteria were

- Routine examination in the mentioned time period (March 2015–April 2015)
- Protocol parameter as described in the following paragraph.

### CT examination

Spiral image acquisition was performed on a second-generation 2 × 64-slice dual source dual energy CT (Somatom Definition Flash, Siemens Healthcare Sector, Forchheim, Germany). Two x-ray tubes, mounted with an angular off-set of 95^o^, rotate around the patient. The scanner offers different scan modes, among two investigated in this study:

1. DECT was performed by using two different tubes voltages (100 kV and tin filtered 140 kV (Sn140 kV), reference tube currents 200/155 mAs) and online dose modulation (CARE Dose 4D, Siemens). The scan was acquired with a detector collimation of 32 × 0.6 mm in craniocaudal direction (pitch 0.6). 3 mm slices were reconstructed using a standard soft tissue reconstruction kernel (standard filtered back projection B31f medium smooth). With a weighting factor of 0.5 the two datasets from the two tubes were fused to virtual images corresponding to a 120 kV scan.
2. SECT was performed with online dose modulation and automatic voltage control (CARE Dose 4D and CarekV, Siemens). The reference tube current was 255 mAs, the reference tube voltage 120 kV. The scan was acquired with a detector collimation of 64 × 0.6 mm in craniocaudal direction (pitch 0.6). 3 mm slices were reconstructed using a standard soft tissue reconstruction kernel (standard filtered back projection B31f medium smooth).

Examination protocol included intravenous application of nonionic iodinated contrast medium (Imeron 300, Bracco, Konstanz, Germany) with a body weight adapted amount and flow rate (see Table 2) via an automated injector. Portal venous images were acquired (bolus-tracking technique) with a scan range from upper abdomen to the inguinal region (approximately 3 cm distal to the symphysis).

**Table 2:** Applied amount of contrast medium^a^

Body weight (kg)	Volume contrast medium^a^ (mL)	Flow rate (mL/s)
<55	85	3.1
55–65	115	3.5
65–90	130	4
>90	145	4.5

^a^Imeron 300, Bracco, Konstanz, Germany

### Data analysis

For each scan, several dosimetry parameters were reported. Most common are the computed tomography dose index (CTDIvol) and the dose length product (DLP). CTDIvol refers to a 32 cm PMMA phantom [32–34]. Although CTDIvol is a useful parameter for characterizing radiation output of the scanner, it is not the patient dose [35]. It underestimates dose for small- and overestimates dose for large patients. To take into consideration patient geometry, the American Association of Physicists in Medicine has introduced size-specific dose estimates (SSDE) [36].

**Fig. 1:**
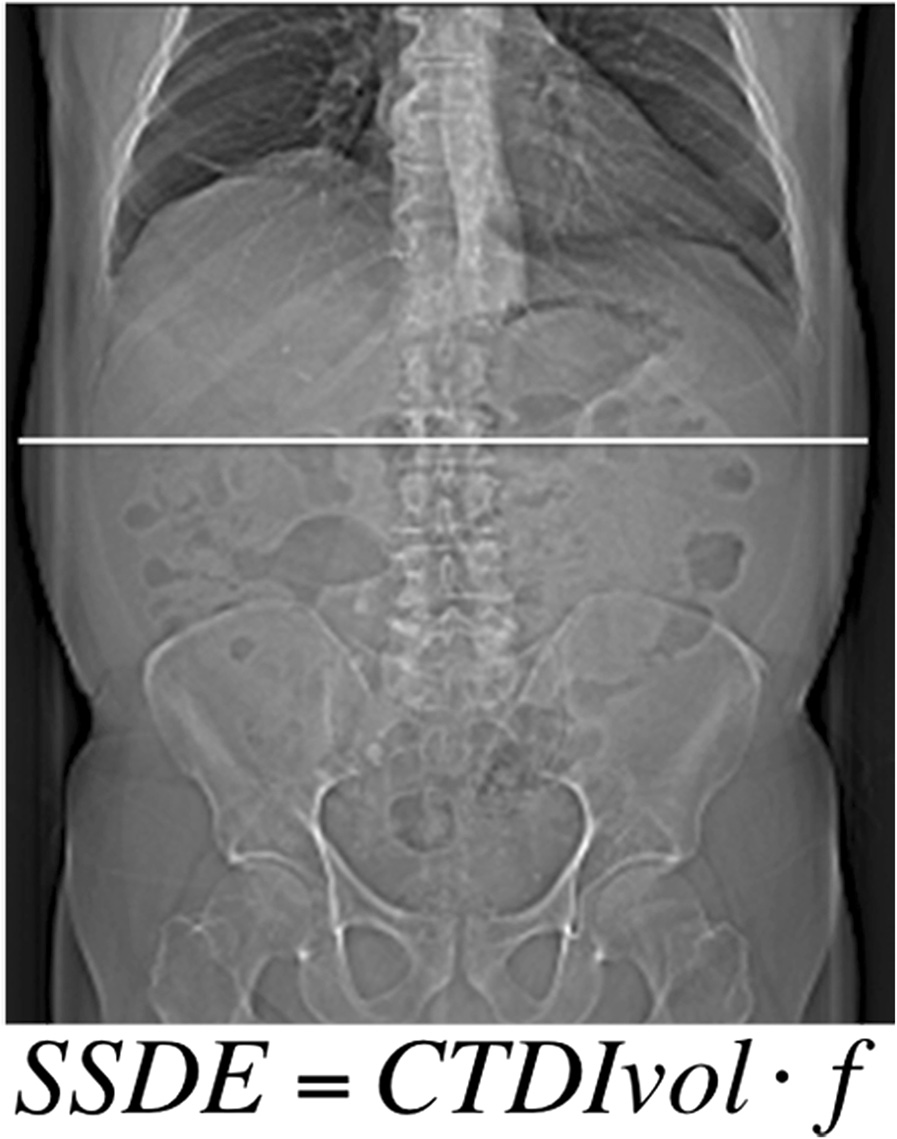
Size-specific dose estimates. Lateral patient diameter was determined on CT-topogram. Based on diameter-dependant conversion factors (f) and CT dose index (CTDIvol), size-specific dose estimates (SSDE) can be calculated

SSDE is based on the diameter of the patient (anterior-posterior, lateral or effective). Diameter can be measured on the patient or determined on a radiograph/CT-topogram. Tables with conversion factors (f) for different diameters between 10 and 45 cm are available. These factors are determined based on data of four research groups working with different methods (phantoms as well as Monte Carlo simulations). CTDIvol given in standard dose protocols can be corrected with the conversion factors by *SSDE* = *CTDIvol* ⋅ *f*. For this study, the maximal lateral diameter of abdomen was determined on CT-topogram (Fig. 1). For 5 patients (3 DECT, 2 SECT) SSDE could not be determined because lateral diameter was above 45 cm and thus not listed in the conversion factor table.

DECT as well as SECT protocols were set up for clinical routine in order to meet the radiologists demands for good image quality at reasonable radiation exposure. The reconstructed images found a broad acceptance in clinical routine for more than 1 year. For this retrospective study, the clinical acceptance was supplemented by the objective parameters image noise and contrast-to-noise ratio, normalized to dose (CNRD).

**Fig. 2:**
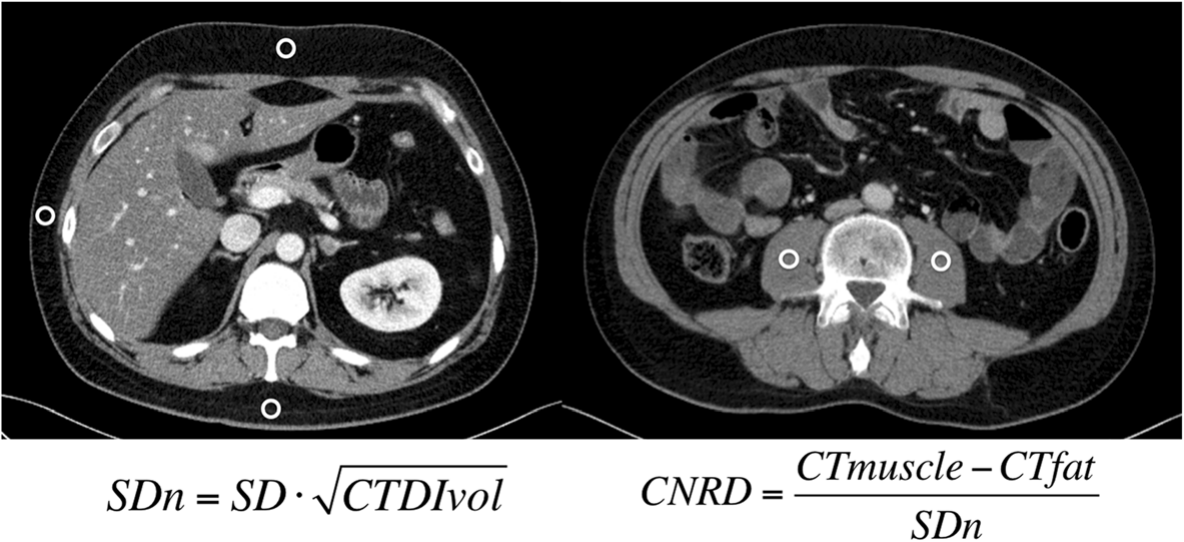
Image noise and contrast-to-noise ratio. *Left:* The image noise (standard deviation (SD) of CT number) was recorded as the mean measurement of three ROIs placed in the subcutaneous fat of anterior, posterior and lateral abdominal wall (CT fat). Image noise was normalized to CTDIvol by $SDn=SD\times \sqrt{CDTIvol}$. *Right:* Mean of CT attenuation of right and left psoas muscle (CTmuscle) was used to calculate the contrast-to-noise ratio (CNRD)

Image noise (standard deviation (SD) of CT number) was recorded as the mean measurement of three ROIs placed in the subcutaneous fat of anterior, posterior and lateral abdominal wall (Fig. 2). For all measurements, the size of the ROI was between 15 and 50 mm^2^. Image noise was normalized because the X-ray generation of the X-ray tube is a random Poisson process. The measured number of photons in the detector will vary from the mean approximately as the inverse square root of the mean number of photons [37], which can be roughly approximated by the inverse square root of CTDIvol. According to this relation, CT noise measured in this study was normalized to SD_n_ by: $SDn=SD\times \sqrt{CDTIvol}$_._

For CNRD, mean CT attenuation numbers of psoas muscle were obtained by placing a circular ROI (15–50 mm^2^) in right and left psoas muscle and calculating the mean of both values (CT_muscle_). Attenuation of muscle tissue was compared to mean attenuation (CT_fat_) and normalized noise of subcutaneous fat by: *CNRD* = (*CTmuscle* − *CTfat*)/*SDn*.

Statistical significance was tested with two-sided *t*-test (significance level α = 0.05). Quantitative variables are expressed as mean with standard deviation.

## Results and discussion

### Results

There was no significant difference (significance level α = 0.05) between the investigated radiation dose surrogates (Table 3):

**Table 3:** Radiation dose and image quality

	CTDIvol (mGy)	DLP (mGy*cm)	SSDE (mGy)	SD_n_ (HU $*\sqrt{\mathrm{mGy}}$)	CNRD (${\mathrm{mGy}}^{-\frac{1}{2}}$)
All	14.2 (±4.2)	674 (±224)	15.9 (±2.2)	44.7 (±14.5)	4.0 (±1.3)
DECT	14.2 (±3.9)	680 (±220)	15.7 (±1.9)	42.2 (±13.9)	3.9 (±1.3)
SECT	14.3 (±4.5)	665 (±231)	16.1 (±2.5)	47.8 (±14.9)	4.0 (±1.3)

Estimated radiation dose of dual energy (DECT) and single energy (SECT) CT scans: Computed tomography dose index (CTDIvol), dose length product (DLP), size-specific dose estimate (SSDE), normalized noise (SD_n_) and normalized contrast to noise ratio (CNRD). Values are given as means (± standard deviation). There was no significant difference for any parameter (*p* < 0.05)

Mean CTDIvol for all scans was 14.2 (±4.2) mGy. Mean CTDIvol for DECT was 14.2 (±3.9) mGy and 14.3 (±4.5) mGy for SECT.

Mean DLP for all scans was 674 (±224) mGy*cm. Mean DLP for DECT was 680 (±220) mGy*cm and 665 (±231) mGy*cm for SECT.

Patient-specific corrections did not change this result:

Mean SSDE was 15.9 (±2.2) mGy for all scans. Mean SSDE for DECT was 15.7 (±1.9) mGy and 16.1 (±2.5) mGy for SECT.

Evaluation of noise, normalized to CTDIvol (SD_n_), revealed no significant difference between DECT and SECT:

Mean SD_n_ for all scans was 44.7 (±14.5) HU $*\sqrt{\mathrm{mGy}}$. Mean SD_n_ for DECT was 42.2 (±13.9) HU $*\sqrt{\mathrm{mGy}}$, versus 47.8 (±14.9) HU $*\sqrt{\mathrm{mGy}}$ for SECT.

Likewise there was no significant difference for CNRD:

Mean CNRD of all scans was 4.0 (±1.3) mGy^− 1/2^. Mean CNRD for DECT was 3.9 (±1.3) mGy^− 1/2^, mean CNRD for SECT was 4.0 (±1.3) mGy^− 1/2^.

**Fig. 3:**
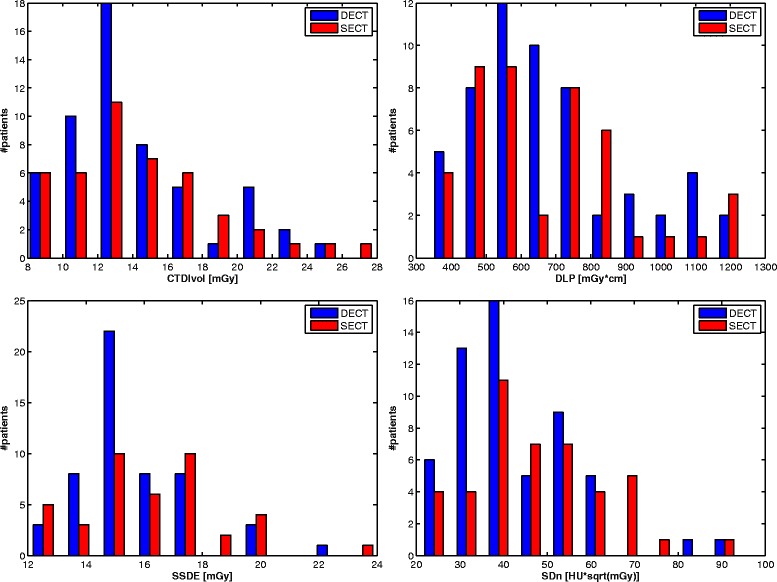
Comparison of radiation dose and image noise. Bar plots of radiation dose parameters and normalized noise (SD_n_). (CTDIvol: computed tomography dose index, DLP: dose length product, SSDE: size-specific dose estimates)

Table 3 summarizes the evaluated dose parameters, Fig. 3 demonstrates a graphical overview of CTDIvol, DLP, SSDE and SD_n_.

As mentioned before, SECT examinations included automatic voltage control. The reference tube voltage of 120 kV was modulated for 39 patients to 100 kV.

### Discussion

Aim of this study was to investigate radiation exposure by DECT abdominal imaging compared to dose-optimized SECT including automatic dose and voltage modulation in clinical routine, considering patient geometries and image quality. Results demonstrate that DECT is feasible without increasing radiation dose or image noise at comparable contrast-to-noise ratios.

The analysis of several parameters underlines this statement, among the commonly accepted DLP and CTDIvol. Mean values from our abdominal examinations (DLP ≤ 680 mGy*cm and CTDIvol ≤ 14.3 mGy) are, following a report from the European Commission, below the most common values in Europe (DLP 800 mGy*cm, CTDIvol 25 mGy) [38].

CTDIvol is a dosimetry parameter that represents the stochastic radiation detriment to a patient, while it is not accounting for different vulnerabilities of tissue [39]. In contrast, the effective dose reflects the relative risk from exposure to ionizing radiation. There are different methods to estimate the effective dose, which were compared in a phantom study using an identical scanner compared to our study [40]. The authors focused on chest CT acquired in different scanning modes (SECT 100 kV, 120 kV, 120 kV high pitch mode and DECT 100/Sn140 kV). Protocols were designed CTDIvol-equivalent compared to the standard 120 kV protocol. The lowest effective dose was observed for the DECT protocol (100/Sn140kV), even compared to the standard SECT 120 kV protocol. Given that this result can be transferred to abdominal imaging, the comparable CTDIvol of DECT and SECT measured in this study could result in a lower effective dose for DECT abdominal scans.

While effective dose reflects tissue vulnerability to ionizing radiation, the specific dose estimate (SSDE) takes into account patient geometry. The corrections based on patient diameter are motivated by the fact, that CTDIvol underestimates dose for small- and overestimates dose for large patients. Consequently, CTDIvol reported for this study was corrected to SSDE, and no significant difference between DECT and SECT was observed. A limitation of this method is, that measuring the diameter of the patient is not standardized. However, measuring the diameter in the centre of the scan length seems to be a very good approximation (mean square difference less than 9 %) compared to slice by slice calculation [41].

Optimizing radiation dose in CT is indispensable, but at the same time image quality must not be affected.

The protocols evaluated for this study were set up for clinical routine in order to meet the radiologists demands for good image quality at reasonable radiation exposure. The broad acceptance of CT images in clinical routine reflects the data of our study: There was no significant difference between DECT and SECT for image noise and CNRD.

Our results go in line with many previous studies comparing radiation dose from DECT and SECT. However, there are some substantial differences in CT protocols and data evaluation compared to our study. In the following, the key points of previous papers as well as the difference to our results shall be described:

Purysko et al. performed a retrospective study in some aspects similar to our study, namely comparing CTDIvol, DLP and SSDE as well as noise of abdomen scans between DECT and SECT. They concluded that DECT imaging of abdomen can achieve noise levels comparable to SECT without a dose penalty [26]. In contrast to our study, information about contrast-to-noise ratio was not provided. Their main limitation was that SECT and DECT were performed on different scanners, which could certainly influence results. Furthermore they did not use automatic voltage control for SECT, which is a substantial difference to our study.

Megibow et al. published a best-practice paper and reported that in their clinical routine, DECT without dose penalty is possible [42]. However, this statement was not underlined with a statistical evaluation.

Schenzle et al. focused on chest examinations. Their phantom study revealed equal effective dose for SECT and DECT in 100/140 kV-mode (2^nd^ generation DECT) [43]. Comparable to our study, image noise was reported similar. The main differences to our study are the anatomical region and the evaluation of DECT with phantoms, not with patients in clinical routine.

In contrary to our results and the mentioned studies above, De Cecco et al. reported a minimal dose increase for DECT [24], without providing any information about image quality or normalization. The reason could be the tube current settings, which were high for DECT (reference 559 mAs for 80 kV tube and 216 mAs for Sn 140 kV tube) compared to SECT (180 mAs for 120 kV).

A recently published phantom study on dose efficiency of DECT suggested a compromised contrast to noise ratio for DECT [31]. The authors state that this result applies specifically to unenhanced soft tissue contrast in a small adult phantom, in contrast to our study setting with contrast enhanced scans in clinical routine.

All studies listed above have in common, that SECT protocols prescribe a fixed tube voltage. But for a fair dose comparison, the optimum dose for a given patient size should be chosen for each examination, which was realized in our study by using automatic voltage control for SECT.

The mentioned studies were, equally to our study, performed with dual source dual energy CTs. In contrast, studies with dual energy techniques based on kV switching show higher doses for DECT [25].

A limitation of this study is that comparisons between DECT and SECT are not within the same patient. In contrast to phantom studies, there is a wide variety of absorption characteristics inevitably coming along with different patient geometries. Considering that the aim of this study was evaluating dose exposure in a clinical routine context, this inhomogeneity had to be accepted. To face this challenge, patient-specific SSDE was calculated. A further limitation is that results refer to specific protocols used in our institution, performed with a dual source CT. There are no comparisons with different CT scanners or modified protocols.

This study showed that DECT abdominal imaging is feasible without increasing radiation dose or deteriorating image quality. Using the higher informational content of DECT data, radiation dose could even be decreased. This is possible by replacing true non-contrast scans with virtual non-contrast scans [44–46]. In addition, CNR can be increased by using nonlinear image blending or advanced monoenergetic reconstruction techniques [47, 48]. In particular for patients requiring frequent follow-up examinations, applying the full spectrum of DECT applications could lead to a significant reduction of radiation dose.

Oncological imaging, a field of increasing socio-economic relevance, can profit from DECT in several ways. Patients need repeated imaging for response monitoring, and DECT can support efforts for radiation dose reduction. Furthermore, the spectral information of DECT data enables tissue characterization and quantification of contrast media. This is essential for monitoring personalized targeted therapies where simple tumour size measurements are not sufficient [49, 50]. DECT offers additional functional information [18, 19], without elevated radiation dose.

## Conclusion

This study suggests that abdominal dual source DECT is feasible without increasing radiation dose or image noise at comparable contrast-to-noise ratios, even compared to SECT exploiting full potential of dose reduction techniques including automatic voltage control. This is of special interest in oncology, where patients require repeated examinations. Thus DECT can contribute to sophisticated oncological imaging without dose penalty.

## Abbreviations

CNRD, dose-normalized contrast-to-noise ratio; DECT, dual energy CT; SD_n_, dose-normalized standard deviation; SECT, single energy CT; SSDE, size-specific dose estimates
